# Primary medical and health preparedness and people's life satisfaction in China: The mediating role of satisfaction with medical and health services

**DOI:** 10.3389/fpubh.2023.1037574

**Published:** 2023-02-06

**Authors:** Zhiwei Tang, Changxiu Ye, Zhuang Fu, Jingran Zhang, Zepeng Gong

**Affiliations:** ^1^School of Public Affairs and Administration, University of Electronic Science and Technology of China, Chengdu, China; ^2^Shenzhen Institute for Advanced Study, University of Electronic Science and Technology of China, Shenzhen, China; ^3^Tianfu Co-innovation Center, University of Electronic Science and Technology of China, Chengdu, China

**Keywords:** primary medical and health preparedness, life satisfaction, satisfaction with medical and health services, China, primary medical and health institutions

## Abstract

**Objective:**

To examine the association between primary medical and health preparedness (PMHP), satisfaction with medical and health services (SMHS), and life satisfaction (LS).

**Methods:**

Using the latest national representative data from the 2019 Chinese Social Survey and the 2018 Health Statistics Yearbook for each province in China, we conducted multi-level models to test the effect of three aspects of PMHP (the number of primary medical and health institutions (PMHIs), the number of beds in PMHIs, and the number of staff in PMHIs) on LS, and the mediation role of SMHS in this effect.

**Results:**

The number of staff in PMHIs has a significant positive effect on people's LS. Besides, this effect is mediated completely by SMHS. However, the number of PMHIs and the number of beds in PMHIs do not affect LS significantly.

**Conclusions:**

PMHP has a positive impact on LS, but this impact is associated only with the number of staff in PMHIs. Therefore, governments should focus on optimizing human resources in PMHIs to meet the LS needs of individuals.

## 1. Introduction

Life satisfaction (LS) refers to the general cognition and evaluation of an individual's satisfaction with their quality of life (1). Individuals with a higher level of LS always exhibit better life outcomes, such as health (2), psychological wellbeing (3), social adjustment (4), and working performance (5), and thus LS is deemed to be an important indicator of the progress of individuals and society in general (6). Therefore, governments around the world regard improving people's LS as one of the core goals of public policies (7, 8). In China, the government has implemented a series of people-centered initiatives in the areas of pensions, employment, health, and social assistance (9, 10). However, reports have shown fluctuations in the LS of Chinese people (11), with a downward trend observed (12). This suggests that these initiatives may not necessarily have a positive association on LS. Therefore, it is critical to understand the effects of people-centered initiatives on LS to provide a reference that can be used to guide the government in devising effective strategies to improve LS.

Since people-centered initiatives are large, macroscopic projects, it is vital that the effects of these initiatives on LS are explored from this perspective. However, previous studies focus mainly on macro-influencing factors such as air quality (13), government spending (14), social justice (15), and urban density (16). As very important people-centered initiatives, medical and health services are major welfare projects provided by governments to improve the level of people's wellbeing (17, 18). Although these services play an important role in health promotion and quality of life, research on the effect of medical and health services on LS has mostly been conducted based on individual's perception (19, 20), while macro-level factors remain a relatively unexplored area.

Limited macro-level studies have mainly studied the impact of medical and health services on LS from an economic perspective. They found that people living in cities with higher health expenditure exhibited better LS (7, 8, 18, 21), namely, increasing expenditures in public health care has a positive effect on citizens' LS. This is because that public health investment can be regarded as a form of social insurance (7), which helps to meet people's medical needs, increase their sense of security and reduce their anxiety related to health problems (8). Moreover, a small number of studies have also explored the impact of medical service characteristics on LS, and found that higher number of health care facilities, better resident-to-employee ratio, and more staff in administration were positive associated with LS (22, 23). Although these studies have made valuable findings, they usually do not consider the heterogeneity of the level of medical institutions in their studies. As a result, it may be difficult to guide the formulation of more precise practical strategies, for example, which level of medical institutions should be invested more.

In China, the institutions providing medical and health services are categorized into three levels (24): level 1 hospitals are primary medical and health institutions (PMHIs), which aim to provide disease prevention and treatment, health promotion, and rehabilitation services to residents in the areas where the institutions are located (25); level 2 hospitals are regional hospitals that provide comprehensive medical and health services to multiple communities and undertake certain teaching and scientific research tasks; level 3 hospitals are regional or national hospitals that provide high-level specialized medical and health services to several regions and performs high-level teaching and scientific research (26, 27). Of these, PMHIs form the foundation of China's medical and health service system and play the role of gatekeeper for people's health. Efficient functioning of PMHIs is considered to be the foundation for achieving the Healthy China strategy. Accordingly, this study aims to explore the effect of primary medical and health preparedness (PMHP), which refers to the preparedness level of PMHIs, on people's LS, and thereby provide implication for policy planners and health care administrators to improve primary medical and health services.

In recent years, and especially after the promulgation of the new medical reform policy in 2009, the Chinese government has attached great importance to the construction of primary medical and health services, and the level of PMHP has been improved significantly. Indeed, the government hopes to address the long-standing problem of “expensive and inaccessible medical services in China,” which has greatly reduced people's LS, by improving the current PMHP level (19). However, we do not know whether an increase in PMHP really improve LS? Therefore, this study will examine the effect of PMHP on LS. To this end, PMHP was evaluated in terms of three aspects in the current study: (1) the number of PMHIs, (2) the number of facilities (i.e., beds) in PMHIs, and (3) the number of staff in PMHIs. This type of measurement can comprehensively reflect the human and financial investment in PMHIs, thus contributing to the continuous quality improvement of delivering health care services. According to previous studies (22, 23), we expect the government's investment in primary health care preparation to be effective in improving LS, and thus assume that people living in areas with higher levels of PMHP show higher levels of LS (**H1**). Moreover, if PMHP positively affect LS, how does this effect work? As we all know, satisfaction with medical and health services (SMHS) is an important indicator of the quality of medical and health services (28, 29) and the basis for the stability of health systems (30). On one hand, SMHS is affected by PMHP. For example, the improvement of PMHP means an increase in the number of staff in PMHIs, while the adequate number of medical personnel was positively correlated with SMHS (31). One the other hand, prior studies have demonstrated that people with higher level of SMHS exhibited better LS (19, 20, 32). Therefore, it can be inferred that SMHS may play a mediation role in the relationship between PMHP and LS. Herein, we hypothesize that the effect of PMHP on LS is mediated by satisfaction with medical and health services (**H2**). This study examined the above hypotheses by matching individual-level data on self-reports of LS and SMHS by respondents of the 2019 Chinese Social Survey (CSS), with provincial-level data on PMHP from the 2018 Health Statistics Yearbook (HSY).

## 2. Methods

### 2.1. Data

The data used in this study were obtained from the 2019 CSS, and the 2018 HSY for each province in China. First, the CSS is a large nationwide survey project launched in 2005 by Institute of Sociology, Chinese Academy of Social Sciences to understand social changes in China (33). The survey was conducted every 2 years. Using a stratified random probability sampling method, the 2019 CSS investigated 10,283 families from 596 villages/communities in 149 counties of 30 provinces of China. The inclusion criteria for eligible participants were aged 18–69 years. The 2019 CSS contained modules for the retrieval of basic information, details of living conditions, social security, and social values as well as social evaluation. The data is publicly available on the CSS official website (http://css.cssn.cn/css_sy/). Considering that the 2019 CSS surveyed respondents' information in 2018, we used the 2018 HSY to obtain data related to PMHP in China. We then combined the data from the 2019 CSS and the 2018 HSY for statistical analysis.

### 2.2. Measurements

#### 2.2.1. Life satisfaction

The question “how satisfied are you with the following items?” was used to measure LS. This question was followed by questions to survey the following six items: education level, leisure/entertainment/cultural activities, social life, family relationship, family economic status, and general LS. The answers for each item ranged from 1 to 10, representing “extremely unsatisfied (score = 1)” to “extremely satisfied (score = 10).” The sum of the scores of the first five items was calculated to evaluate LS with higher scores indicating a higher level of LS. In this study, the Cronbach's alpha for LS was 0.746. Moreover, the score of the last item (i.e., general LS) was used to conduct robustness analysis.

#### 2.2.2. Primary medical and health preparedness

To objectively reflect the PMHP level of each province, we divided the data of each province on the three aspects of PMHP (i.e., the number of PMHIs, the number of beds in PMHIs, and the number of staff in PMHIs) by the population of each province to obtain the number of three aspects per 10,000 people at the provincial level. A province with a higher number per 10,000 people indicates that it has a higher level of PMHP.

#### 2.2.3. Satisfaction with medical and health services

The question “how do you think of the medical and health services provided by governments?” was used to evaluate the level of SMHS. The response options included “very bad (score = 1),” “not good (score = 2),” “good (score = 3),” “very good (score = 4),” and “unclear.” In this study, the “unclear” option was regarded as a missing value.

#### 2.2.4. Satisfaction with medical security

Satisfaction with medical security (SMS) was used as the proxy variable of SMHS for robustness analysis. SMS was evaluated by the question “how satisfied are you with the medical security provided by governments?” with 11 response options ranging from “extremely unsatisfied (score = 1)” to “extremely satisfied (score = 10),” and one “unclear” option. The “unclear” option in this study was regarded as a missing value.

#### 2.2.5. Social demographics

The following social demographic information was retrieved for each participant: sex (female = 0, male = 1), age, marital status, education level, economic status, and religious belief (or not); these characteristics were used as control variables (8, 34–36). Age was used as a continuous variable. Marital status was recorded as five categories: “unmarried,” “married,” “divorced,” “widowed,” and “cohabiting.” Education level was considered as a continuous variable categorized as “illiterate (score = 1),” “primary (score = 2),” “middle (score = 3),” “high school or equivalent (score = 4),” “college (score = 5),” “bachelor's degree (score = 6),” and “master's degree or above (score = 7).” Economic status was evaluated by the question “what do you think of your social-economic status in the local area?” with the following response options: “low (score = 1),” “lower medium (score = 2),” “medium (score = 3),” “upper medium (score = 4),” “high (score = 5),” and “hard to say,” which was coded as a missing value. Whether a respondent had a religious belief was recoded as a dummy variable, with having a religious belief coded as 1, and 0 otherwise.

### 2.3. Statistical analysis

First, the frequency (percentage) or mean (standard deviation) of variables was reported. We then mapped the provincial distribution of PMHP levels. Next, we used a multi-level (two-level) model for statistical analysis since our data included individual level and provincial level data. We estimated the effect of PMHP (X) on LS (Y) and whether such effect was mediated by SMHS (M). In detail, we analyzed data according to Baron and Kenny's test for mediation effect (37) using the following four models: (1) the effect of X on Y (model 1), (2) the effect of X on M (model 2), (3) the effect of M on Y when adjusted for X (model 3), and (4) the effect of X on Y when adjusted for M (model 4). Furthermore, we conducted a series of robustness tests, including replacing the dependent variable with general LS (step 1) and replacing both the dependent variable with general LS and the mediating variable with SMS (step 2). *P* < 0.05 was considered statistically significant. All analyses were performed using Stata/MP 14.0.

## 3. Results

Of the participants, 57.08% were females and 13.10% had a religious belief (Table 1). The mean age of the sample was 46.59 years, and most of them were married (80.38%). The mean scores for education level and economic status were 3.243 and 2.341, respectively. Participants showed moderate to high levels of satisfaction with medical security (mean = 6.735) and medical and health services (mean = 3.022). The mean scores for LS and general LS were 32.053 (standard deviation = 8.834) and 7.091 (standard deviation = 2.234), indicating participants had a relatively positive attitude toward their life.

**Table 1:** Characteristics of the study sample.

	**Frequency/ mean (SD)**	**Percent/ range**	***N***
Sex^+^			10,283
Female	5,870	57.08	
Male	4,413	42.92	
Marriage status^+^			10,278
Unmarried	1,260	12.26	
Married	8,261	80.38	
Divorced	290	2.82	
Widowed	443	4.31	
Cohabiting	24	0.23	
Having a religious belief^+^			10,283
No	8,936	86.90	
Yes	1,347	13.10	
Age (years)^§^	46.59 (14.25)	18–69	10,283
Economic status^§^	2.341 (0.927)	1–5	10,134
Education level^§^	3.243 (1.414)	1–7	10,264
SMS^§^	6.735 (2.617)	1–10	9,899
SMHS^§^	3.022 (0.720)	1–4	9,725
LS^§^	32.05 (8.834)	5–50	10,282
General LS^§^	7.091 (2.234)	1–10	10,282

^+^Frequency and percent are reported;

§ Mean (SD) and range are reported.

SD, standard deviation; LS, life satisfaction; SMHS, satisfaction with medical and health services; SMS, satisfaction with medical security.

**Figure 1:**
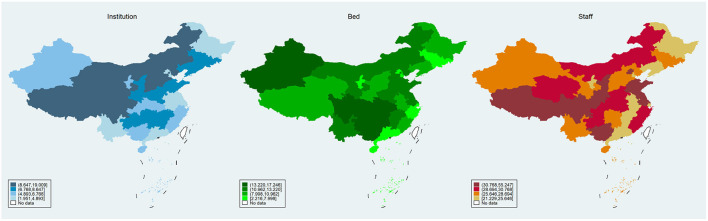
Distribution of provincial primary medical and health preparedness. **(Left, middle, and right)** Show the distribution of PMHIs (number per 10,000 people, the number of beds in PMHIs per 10,000 people, and the number of staffs in PMHIs per 10,000 people, respectively).

Figure 1 exhibits the provincial distribution of the PMHP level. The median number of PMHIs per 10,000 people was 6.768 [interquartile range (IQR): (4.89, 8.647)]. The median number of beds in PMHIs was 10.962 [IQR = (7.998, 13.220)], and the median value for the number of staff in PMHIs was 28.694 [IQR = (25.646, 30.768)]. According to these results, one bed in PHMIs serves almost 1,000 people, and one member of staff in PMHIs serves nearly 300 people.

The relationships between PMHP, SMHS, and LS are shown in Table 2. In model 1, the number of PMHIs and the number of beds in PMHIs were not significantly associated with LS, while the number of staff in PMHIs showed a significant positive effect on LS (*β* = 0.107, *P* < 0.05). Similarly, the analysis using in model 2 showed a significant correlation only between the number of staff in PMHIs and SMHS (*β* = 0.015, *P* < 0.001). After incorporating both PMHP and SMHS into the model, the relationship between SMHS and LS was significant (*β* = 1.792, *P* < 0.001), although the effects of all the three aspects of PMHP on LS were not significant. According to these results, SMHS has a full mediation effect on the association between the number of staff in PMHIs and LS.

**Table 2:** Associations between primary medical and health preparedness, satisfaction with medical and health service, and life satisfaction.

	**Model 1**	**Model 2**	**Model** ** 3/4**
	**LS**	**SMHS**	**LS**
PMHP			
Number of PMHIs	−0.082 (0.090)	−0.003 (0.008)	−0.079 (0.085)
Number of beds in PMHIs	−0.096 (0.055)	−0.004 (0.005)	−0.093 (0.052)
Number of staff in PMHIs	0.107^*^ (0.047)	0.015^***^ (0.004)	0.082 (0.044)
SMHS			1.792^***^ (0.111)
Sex (male)	0.452^**^ (0.162)	−0.027 (0.015)	0.456^**^ (0.164)
Age (years)	0.080^***^ (0.007)	0.002^*^ (0.001)	0.079^***^ (0.008)
Education level	1.463^***^ (0.069)	−0.045^***^ (0.006)	1.525^***^ (0.070)
Economic status	3.219^***^ (0.085)	0.063^***^ (0.008)	3.115^***^ (0.087)
Having a religious belief (yes)	−0.110 (0.242)	−0.032 (0.022)	−0.177 (0.244)
Marriage status (ref: unmarried)			
Married	−2.928^***^ (0.294)	−0.108^***^ (0.027)	−2.754^***^ (0.295)
Divorced	−4.658^***^ (0.537)	−0.074 (0.050)	−4.350^***^ (0.550)
Widowed	−1.928^***^ (0.505)	−0.091 (0.047)	−1.790^***^ (0.516)
Cohabiting	−0.435 (1.682)	0.039 (0.160)	−0.733 (1.741)
*N*	10,110	9,594	9,594

PMHIs, Primary medical and health institutions; LS, life satisfaction; SMHS, satisfaction with medical and health services.

* *P* < 0.05,

** *P* < 0.01,

*** *P* < 0.001.

The results of the robustness tests are shown in Table 3. Whether replacing only the dependent variable (step 1) or both the dependent and mediating variables (step 2), the results demonstrated that only the number of staff in PMHIs affected LS positively. Furthermore, this effect was fully mediated by SMHS. These findings indicate that the associations between all the three aspects of PMHP and LS were robust.

**Table 3:** Robustness tests.

	**Step 1**		**Step 2**	
	**General LS**	**General LS**	**SMS**	**General LS**
Number of PMHIs	0.010 (0.025)	0.014 (0.023)	0.022 (0.029)	0.006 (0.022)
Number of beds in PMHIs	−0.020 (0.015)	−0.018 (0.014)	−0.015 (0.018)	−0.017 (0.013)
Number of staffs in PMHIs	0.026^*^ (0.013)	0.017 (0.012)	0.051^***^ (0.015)	0.014 (0.011)
SMHS		0.443^***^ (0.029)		
SMS				0.219^***^ (0.008)
*N*	10,110	9,594	9,763	9,763

PMHIs, Primary medical and health institutions; LS, life satisfaction; SMHS, satisfaction with medical and health services; SMS, satisfaction with medical security; adjusted for all control variables.

* *P* < 0.05;

*** *P* < 0.001.

## 4. Discussion

To the best of our knowledge, this is the first study to examine the impact of PMHP on people's LS from a macro perspective. We measured PMHP in terms of the number of PMHIs, the number of beds in PMHIs, and the number of staffs in PMHIs. Using nationally representative data in China, we analyzed the direct impacts of the three aspects of PMHP on LS, and the indirect effects of PMHP on LS mediated through improved satisfaction with medical and health services. We have obtained some interesting and novel findings.

In this study, the number of staff in PMHIs was shown to play a significant positive role in improving people's LS. Specifically, people living in provinces with more primary medical and health workers per 10,000 people exhibit a higher level of LS. This is similar to previous findings, which have shown that the better resident-to-staff ratio is associated with higher quality of life (22). Furthermore, the effect of the number of staff in PMHIs on LS is fully mediated by SMHS. In other words, the number of staff in PMHIs does not directly influence people's LS, but affects LS by affecting people's satisfaction with medical and health services. This finding is understandable because a better resident-to-staff ratio means that each employee manages fewer patients, which means less stress for employees (38), and they can better serve patients and meet their needs (39), resulting in better patient satisfaction with medical and health services (30, 40). This in turn translates into better perceptions of health status and life satisfaction (32, 41). Moreover, more staff in PMHIs results in a better experience of obtaining medical and health services (i.e., accessibility), such as increased opportunities and reduced waiting time to obtain services, with the accessibility of medical and health services being an important determinant of LS (19, 20, 42). Accordingly, this study highlights the importance of human resources in the construction of primary medical and health services (43). Indeed, human resources have an critical role in the development of health systems (44, 45) and can improve the performance of healthcare institutions in terms of providing medical and health services (46). Sheikhbardsiri et al. (47) concluded that it should settle the shortage of human resources in health services, because human resource supply is one of the most vital factors in achieving institutional goals and the most valuable factor in the producing and delivering of services (48). Therefore, this study suggests that initiatives to strengthen PMHPs should start by increasing the number of staff, such as general practitioners, in PMHIs.

Surprisingly, the other two aspects of PMHP (i.e., the number of PMHIs and the number of beds in PMHIs) have no significant effect on LS, which means that these aspects not only have no direct effect on LS, but also do not influence LS by affecting satisfaction with medical and health services. There results are inconsistent with prior studies that showed that residents living in places with more care institutions and facilities exhibited better wellbeing (23). Therefore, in contrast to previous research (49), our study does not support increasing the number of PMHIs as a strategy for developing primary medical and health services. Indeed, there are 970,000 PMHIs in China, basically achieving full coverage of urban and rural communities (50). Furthermore, our findings do not suggest that increasing the number of beds in PMHIs will improve LS. Currently, the phenomenon of empty beds in China's PMHIs indicates that the supply of beds exceeds demand. Therefore, it is important to improve the utilization rate of beds rather than increasing the number of beds. This, however, is beyond the scope of the present study.

Some limitations of this study should be noted. First, PMHP encompasses a wide range of elements, but this study was focused on only three aspects: the number of PMHIs, the number of beds in PMHIs, and the number of staff in PMHIs. Future research could be conducted to explore the impact of other aspects of PMHP (e.g., service capacity, funding, and staff structure) on LS. Second, the number of staff in PMHIs corresponds to the total number of doctors, nurses, pharmacists, and other employees; however, due to the limitation of data acquisition, we were unable to confirm which type of staff had a greater impact on LS. Third, although many factors affect LS, we included only socio-demographics as control variables and omission variables may lead to biased results.

## 5. Conclusion

In conclusion, PMHP has a positive impact on LS, but this impact is derived only from the number of staff in PMHIs, and not the number of PMHIs or the number of beds in PMHIs. Moreover, the effect of the number of staff in PMHIs on LS is indirect and must be mediated by satisfaction with medical and health services. This study suggests that the government should focus on the construction of human resources in PMHIs.

## Data availability statement

Publicly available datasets were analyzed in this study. This data can be found here: the data of the 2019 Chinese Social Survey (http://css.cssn.cn/css_sy/) and the 2018 Health Statistics Yearbook for each province in China (http://www.nhc.gov.cn/mohwsbwstjxxzx/tjtjnj/202106/ff9efb87ead24385b83ddb9eb0e3df5f.shtml).

## Ethics statement

Ethical review and approval was not required for the study on human participants in accordance with the local legislation and institutional requirements. Written informed consent for participation was not required for this study in accordance with the national legislation and the institutional requirements.

## Author contributions

ZG and ZT designed and wrote the first draft of the paper and conducted the data analysis. CY and ZF were major contributors of data collection and paper revision. JZ made contributions to data interpretation and paper revision. All authors have read and approved the final version.
